# Targeting oncogene-induced cellular plasticity for tumor therapy

**DOI:** 10.1007/s44307-024-00030-y

**Published:** 2024-07-15

**Authors:** Bin Li, Lingling Zheng, Jianhua Yang, Lianghu Qu

**Affiliations:** 1 MOE Key Laboratory of Gene Function and Regulation, State Key Laboratory of Biocontrol, School of Life Sciences, Sun Yat-sen University, Guangzhou, 510275 Guangdong China; 2 The Fifth Affiliated Hospital, Sun Yat-sen University, Zhuhai, 519000 China

**Keywords:** Cellular plasticity, Oncogene, EMT, Metastasis, Precision medicine

## Abstract

Cellular plasticity, the remarkable adaptability of cancer cells to survive under various stress conditions, is a fundamental hallmark that significantly contributes to treatment resistance, tumor metastasis, and disease recurrence. Oncogenes, the driver genes that promote uncontrolled cell proliferation, have long been recognized as key drivers of cellular transformation and tumorigenesis. Paradoxically, accumulating evidence demonstrates that targeting certain oncogenes to inhibit tumor cell proliferation can unexpectedly induce processes like epithelial-to-mesenchymal transition (EMT), conferring enhanced invasive and metastatic capabilities. In this review, we summarize the latest models elucidating the biology of oncogenes that concurrently promote cell proliferation while inhibiting metastasis. We suggest that the complexity of oncogene-induced cellular plasticity, involving the participation of multiple signaling pathways and mechanisms, necessitates a multifaceted approach, prompting a shift towards precision targeting strategies that can effectively target oncogenes without exacerbating metastatic potential.

## Introduction

Cancer is a complex and heterogeneous disease characterized not merely by uncontrolled cell growth but also by the remarkable adaptability of cancer cells to survive under various stress conditions (Hanahan 2022; Swanton et al. 2024; Zhang et al. 2024). This adaptability, termed cellular plasticity, is a fundamental hallmark of cancer cells that significantly contributes to treatment resistance, tumor metastasis, and disease recurrence (Pérez-González et al. 2023). Cellular plasticity is a major driving force behind tumor heterogeneity, where distinct subpopulations of cancer cells coexist within the same tumor, exhibiting different phenotypic and functional properties, such as highly proliferative or metastatic phenotypes (Gupta et al. 2019). This heterogeneity poses a significant obstacle to effective cancer treatment, as it increases the likelihood of treatment-resistant subpopulations emerging and contributing to disease relapse.

Oncogenes, the driver genes that promote uncontrolled cell proliferation, are have long been recognized as key drivers of cellular transformation and tumorigenesis (Felsher 2003). Therefore, researchers have developed diverse strategies targeting oncogenes at DNA, RNA, and protein levels to combat cancer, including genome editing, RNA interference, small molecule inhibitors, and immunotherapies. This multi-pronged approach aims to provide comprehensive and personalized cancer treatments by targeting oncogene expression, activity, and signaling pathways, potentially overcoming resistance mechanisms and improving patient outcomes. Nevertheless, accumulating evidence demonstrates that the activation of certain oncogenes can induce processes like epithelial-to-mesenchymal transition (EMT) in tumor cells, conferring enhanced invasive and metastatic capabilities (Grunert et al. 2003). Paradoxically, some studies have revealed that targeting oncogenes to inhibit tumor cell proliferation can unexpectedly promote metastasis. Oncogenes, such as *CREB1* (cAMP Response Element Binding Protein 1), *MYC* (MYC Proto-Oncogene, bHLH Transcription Factor), and so on, can hijack and dysregulate various cellular pathways and processes, leading to the induction of cellular plasticity (Li et al. 2022; Liu et al. 2012). These oncogenes can activate transcriptional programs that govern EMT, stem cell properties, and metabolic reprogramming, all of which contribute to the acquisition of diverse and resilient cancer cell states. This phenomenon underscores the importance of considering tumor cell plasticity and its influence on the biological behavior of the tumor when designing and implementing tumor treatment strategies.

In this review, we will focus on the core concept of "targeting oncogenes induces a phenotypic shift between proliferation and metastasis", and investigate into the biology of oncogenes that promote cell proliferation but inhibit metastasis, explore the rationale behind combination therapies, and discuss the current strategies and emerging directions in the field. By conducting an in-depth analysis of the available evidence and exploring the potential of these innovative approaches, we aim to provide a comprehensive overview of the state of the art in targeting complex oncogenes and improving cancer treatment outcomes.

## Mechanisms of oncogene-induced cellular plasticity

### Overview of cellular plasticity in *cancer*

**Fig. 1:**
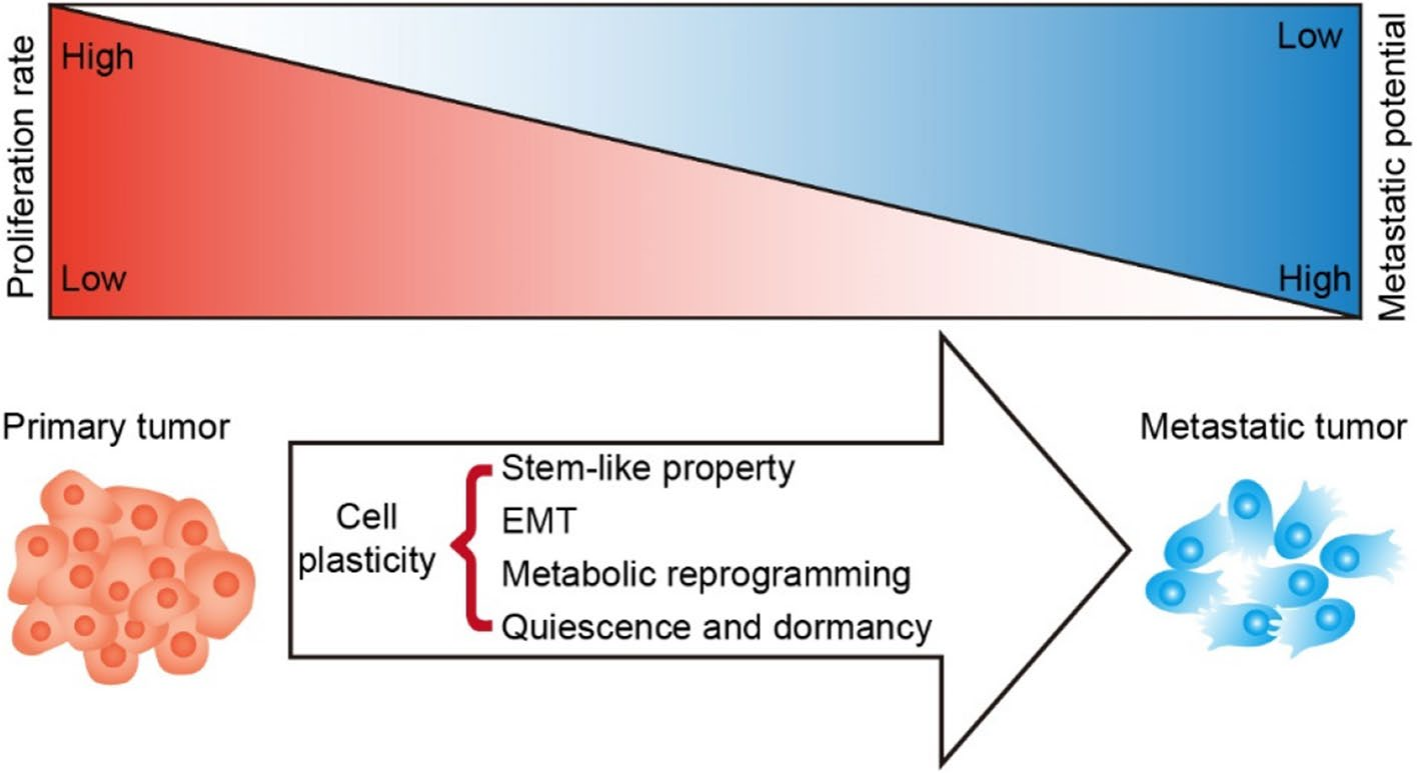
Changes of proliferation rate and metastatic potential during tumor progression

Cellular plasticity in cancer encompasses a wide range of phenotypic and metabolic adaptations that allow cancer cells to survive and thrive in various microenvironmental conditions. This plasticity can manifest in different forms, including stem-like properties, EMT, metabolic reprogramming and quiescence and dormancy (Bergers et al., 2021; Brabletz 2012; Pérez-González et al. 2023; Schwitalla 2014). These states result from a dynamic balance between proliferative and metastatic phenotypes (Fig. 1).

Cancer cells can acquire stem cell-like characteristics, such as self-renewal capacity, multi-lineage differentiation potential, slow proliferation rate and increased resistance to therapeutic interventions (Huyghe et al. 2024). These cancer stem cells (CSCs) are believed to play a crucial role in tumor metastasis, and disease recurrence. EMT is a process through which epithelial cells lose their cell-cell adhesion and polarity, transitioning into a more invasive and migratory mesenchymal phenotype (Brabletz et al. 2018). EMT has been implicated in cancer metastasis, as well as the acquisition of stem-like properties and treatment resistance. Cancer cells can rewire their metabolic pathways to adapt to nutrient-deprived or hypoxic conditions within the tumor microenvironment (Bergers et al., 2021). This metabolic plasticity involves shifts in energy production pathways, such as increased glycolysis, glutaminolysis, or oxidative phosphorylation, enabling cancer cells to meet their biosynthetic and energetic demands under stress. In response to specific environmental cues or therapeutic interventions, cancer cells can enter a quiescent or dormant state, characterized by reduced proliferation and metabolic activity (Giancotti 2013). This cellular plasticity allows cancer cells to persist in a treatment-resistant state, potentially contributing to tumor recurrence and metastasis at later stages.

The remarkable ability of cancer cells to undergo these diverse phenotypic and metabolic transitions is a fundamental characteristic of cellular plasticity, enabling tumor cells to adapt, survive, and propagate in various microenvironmental contexts.

### Oncogenes involved in inducing cellular plasticity

**Fig. 2:**
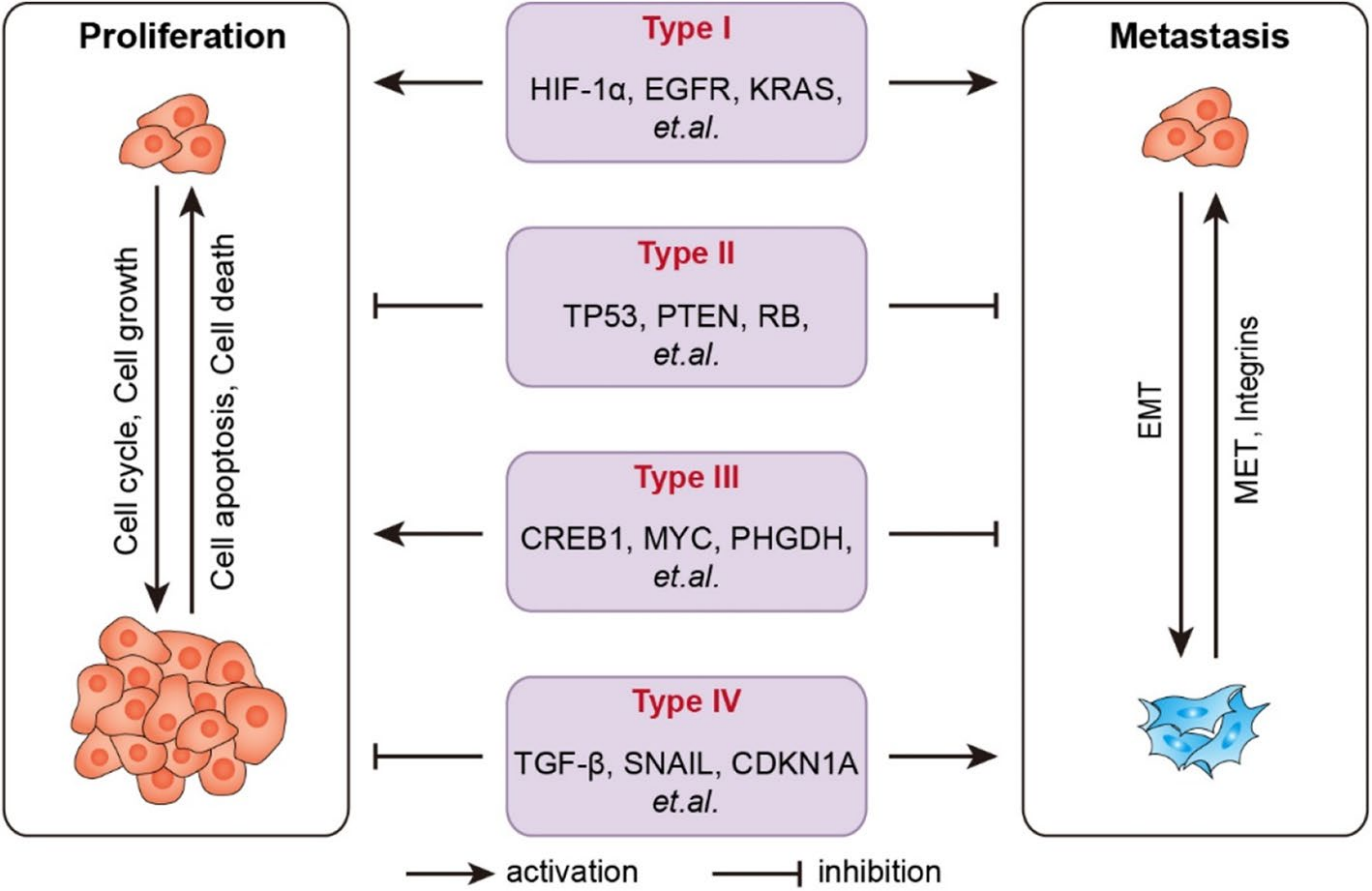
The regulatory pattern of genes in cell proliferation and metastasis

The regulatory pattern of genes in cell proliferation and metastasis is a sophisticated orchestration of molecular events that determine the behavior of cancer cells. This intricate balance is characterized by the dual roles’ genes can play in either promoting or inhibiting these processes. Based on the pattern of genes simultaneously regulating cell proliferation and metastasis, genes can be classified into four types. The first category, Type I genes, often oncogenes, are well-known for their role in driving cellular transformation, with numerous studies documenting their ability to promote both cell proliferation and metastasis (Buscail et al. 2020; Ciardiello et al., 2008; Gilkes et al. 2014) (Fig. 2, Type I). In contrast, Type II genes function as inhibitors for both processes (Fig. 2, Type II). Typically, these are tumor suppressor genes that are crucial in maintaining normal cell cycle control and preventing the development of metastatic capabilities in cancer cells. Meanwhile, accumulating evidence suggests that certain oncogenes also play a critical role in inducing and maintaining cellular plasticity in cancer cells by regulating the switch between proliferation and metastasis. Specifically, Type III genes possess the remarkable capacity to enhance cell proliferation while concurrently restraining cell metastasis (Li et al. 2022; Liu et al. 2012; Rossi et al. 2022) (Fig. 2, Type III). Lastly, Type IV genes are those that inhibit cell proliferation but, in a counterintuitive manner, promote metastasis (Fig. 2, Type IV).

In these types of genes, the oncogenic properties of Type III genes are particularly noteworthy. Inhibiting these genes, though effective in suppressing tumor cell proliferation, exacerbates the fatal metastatic potential of tumor cells. Many research has found that certain oncogenes mainly regulate tumor metastasis by negatively regulating EMT and integrin-related processes.

#### Oncogenes promote proliferation while inhibiting metastasis by regulating EMT

**Fig. 3:**
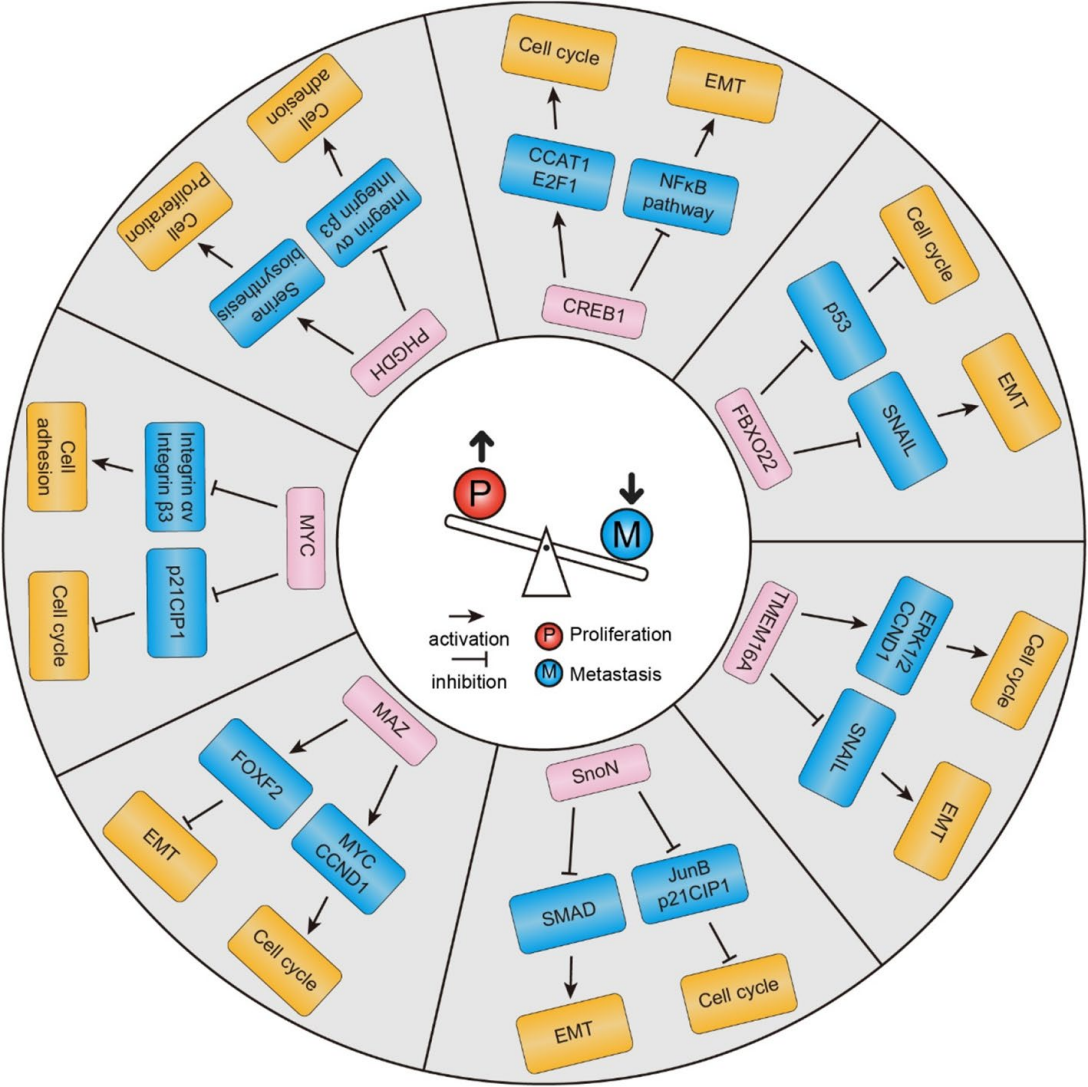
The mechanisms by which oncogenes can concurrently regulate both cell proliferation and metastasis

EMT is a process enabling epithelial cells to undergo profound alterations in morphology and behavior, culminating in the acquisition of a migratory and invasive phenotype, characteristic of cancer metastasis. While numerous oncogenes have been identified as enhancers of tumor metastasis through the promotion of EMT, a subset of oncogenes, such as *CREB1*, *MYC* and *FBXO22* (F-box protein 22), has been observed to concurrently facilitate proliferation while inhibiting EMT (Fig. 3). This underscores the intricate and occasionally contradictory roles these oncogenes play in tumor biology.

CREB1, a transcription factor with pleiotropic functions, exemplifies this duality (Li et al. 2022). In colorectal cancer, CREB1 activates the transcription of *CCAT1* (colon cancer associated transcript 1), which upregulates *MYC*, a potent oncogene. This axis drives cell cycle progression and enhances cell proliferation. Yet, CREB1 also suppresses the NF-κB (Nuclear factor kappa B) pathway, a key regulator of EMT, thereby inhibiting the migratory and invasive capabilities of cancer cells (Fig. 3). Additionally, as a pivotal responder to the second messenger cAMP, CREB1 potentially contributes to metastasis inhibition through the cAMP-PKA-CREB1 signaling cascade. For example, elevated intracellular cAMP levels, along with subsequent PKA activation, induce a mesenchymal-to-epithelial transition (MET) in mesenchymal human mammary epithelial cells by phosphorylating histone demethylase PHF2 (PHD Finger Protein 2) (Pattabiraman et al. 2016). In pancreatic cancer cells, Zimmerman et al. demonstrated that cAMP-elevating drugs hindered both basal and TGF-β-directed pancreatic ductal adenocarcinoma (PDAC) cell migration and invasion (Zimmerman et al. 2015). These findings underscore the pivotal role of CREB1 in maintaining the delicate balance between tumor growth and metastatic potential.

Similarly, FBXO22, a component of the SCF ubiquitin ligase complex, promotes cell proliferation in breast cancer, particularly in triple-negative breast cancer cells (Sun et al. 2018). Its high expression levels are associated with better clinical outcomes, suggesting an anti-metastatic role. FBXO22 inhibits EMT by regulating the degradation of the SNAIL (Snail Family Transcriptional Repressor 1) protein, a key regulator of EMT. A mutation in FBXO22, such as the W52R, disrupts its function, leading to increased metastasis, underscoring the oncogene's dual role in cancer progression (Fig. 3). Additionally, FBXO22 may facilitate cell cycle progression by mediating the degradation of p53, contributing to its role in cell proliferation (Johmura et al. 2016).

TMEM16A (Transmembrane member 16A), an ion channel protein, also plays a dual role in squamous cell carcinoma of the head and neck. It promotes cell growth through the RAS-RAF-ERK-CCND1 pathway (Duvvuri et al. 2012) but is downregulated in metastatic lymph nodes, where its loss is associated with increased cell motility and invasiveness (Fig. 3) (Shiwarski et al. 2014). TMEM16A's interaction with the actin-scaffolding protein Radixin affects the epithelial characteristics of the cell and its ability to undergo EMT by regulating *SNAIL*, further illustrating the complex interplay between tumor cell growth and metastasis.

*SnoN* (Ski-related novel protein N), a member of the Ski family of proto-oncogenes, is recognized as a negative regulator of TGF-β signaling by repressing the activity of Smad proteins (Zhu et al. 2007). High levels of SnoN have been observed in various human cancer cell lines, and this elevated expression has been linked to its pro-oncogenic role (Fig. 3). SnoN promotes mitogenic transformation of breast and lung cancer cell lines in vitro and tumor growth in vivo through downregulating *JunB* and *CDKN1A* (Cyclin Dependent Kinase Inhibitor 1A, also known as p21CIP1), suggesting that it may facilitate the establishment of primary tumor colonies by enhancing cell growth. Despite its role in promoting tumor growth. SnoN also exhibits antitumorigenic properties by inhibiting EMT and subsequent metastasis, involving both Smad-dependent and Smad-independent pathways. This multifaceted regulation allows SnoN to maintain a delicate balance between tumor growth and the metastatic potential of cancer cells.

Lastly, MAZ (Myc-associated zinc finger protein), a transcription factor, promotes cell proliferation by activating genes involved in cell cycle progression through transcriptional regulation of several oncogenes such as *MYC* (Bossone et al. 1992) and *CCND1* (Cyclin D1) (Wang et al. 2024). In the context of breast cancer, MAZ expression is elevated in cancer tissues compared to normal breast tissues, particularly in highly proliferative subtypes such as luminal B and HER2-enriched cancers. Experimental evidence suggests that MAZ expedites cell proliferation, with its overexpression linked to increased cell growth and colony formation in basal-like breast cancer (BLBC) cells (Yu et al. 2017). Despite its role in promoting proliferation, MAZ also exhibits anti-metastatic properties (Fig. 3) (Yu et al. 2017). Low MAZ expression levels in BLBC patients correlate with shorter distant metastasis-free survival (DMFS). Mechanistically, MAZ binds to the GC-rich promoter region of *FOXF2* (Forkhead box protein F2) and activates its transcription. FOXF2, in turn, has been demonstrated to suppress the EMT program and the aggressive behavior of BLBC cells through regulation of *TWIST1* (Twist family bHLH transcription factor 1). Thus, the MAZ-FOXF2 axis represents a critical regulatory pathway controlling both the proliferation and metastasis of BLBC.

In conclusion, the ability of oncogenes to promote cell proliferation while inhibiting metastasis by regulating EMT highlights the intricate balance in tumor biology. These findings underscore the importance of understanding the complex roles of oncogenes in cancer progression. Therapeutic strategies that target these oncogenes and their regulatory pathways could potentially enhance tumor cell proliferation while curbing metastatic potential, offering a promising avenue for the development of more effective cancer treatments.

#### Oncogenes promote proliferation while inhibiting metastasis by regulating integrin

The interplay between oncogene expression and integrins, which are critical mediators of cell adhesion, migration, and invasion, represents a complex and nuanced relationship in tumor biology. Notably, certain oncogenes, including *MYC* and *PHGDH* (Phosphoglycerate Dehydrogenase), have been documented to facilitate cell proliferation while concurrently suppressing metastasis via the modulation of integrins (Fig. 3).

*MYC*, a transcription factor-encoding oncogene, is widely recognized for its role in driving tumor cell proliferation and metabolic reprogramming across various cancer types. Its ability to stimulate cell division is well-established; however, recent findings have revealed a paradoxical function of *MYC* in inhibiting metastasis (Fig. 3) (Liu et al. 2012). Specifically, MYC has been shown to directly suppress the transcription of αv and β3 integrin subunits, which are integral to cell motility, invasion, and metastasis. This duality is exemplified in breast cancer cells, where MYC overexpression results in enhanced proliferation but also reduced cell motility and invasiveness, thereby limiting metastatic potential. This suggests that while targeting MYC to control tumor growth is a viable strategy, it must be approached with caution due to the potential risk of promoting metastasis.

PHGDH, an enzyme involved in cancer cell metabolism, is another oncogene with a dual role in tumor biology. Its catalytic activity is crucial for promoting cancer cell proliferation by increasing de novo serine biosynthesis (Possemato et al. 2011). However, recent research has uncovered a more complex function for PHGDH in modulating the metastatic process (Fig. 3) (Rossi et al. 2022). Low or heterogeneous PHGDH expression within tumors is associated with increased metastatic dissemination, indicating that PHGDH levels may act as a regulatory switch between proliferative and metastatic phenotypes. Mechanistically, the loss of PHGDH activates the hexosamine-sialic acid pathway, leading to aberrant protein glycosylation and increased sialylation of integrin αv and β3, which enhances cell migration and invasion. This metabolic reprogramming induced by PHGDH loss contributes to a pro-metastatic phenotype, despite its role in supporting cell proliferation.

The intricate balance between the proliferative and metastatic roles of these oncogenes underscores the complexity of tumor progression. The regulation of integrin proteins by MYC and PHGDH provides a valuable insight into how oncogenes can modulate tumor cell plasticity. Understanding these mechanisms is essential for the development of targeted therapies that can effectively control tumor growth and limit cancer spread.

In conclusion, the intricate mechanisms by which oncogenes regulate cellular plasticity in cancer underscore the complexity of tumor biology. While traditionally known for promoting cell proliferation, certain oncogenes exhibit a dual role by simultaneously inhibiting metastasis. This paradoxical behavior is exemplified through their regulation of processes such as EMT and integrin signaling. By understanding and targeting these oncogenic pathways, there is potential to develop more effective cancer treatments that prioritize inhibiting metastasis while controlling tumor growth. While some oncogenes are known to promote cell proliferation and inhibit metastasis, there are instances where certain genes, such as *TGF-β* (Huang et al. 2018), *SNAIL* (Vega et al. 2004), and *CDKN1A* (Qian et al. 2013), exhibit the opposite behavior, inhibiting cell proliferation while simultaneously promoting metastasis. Targeting these genes could offer novel therapeutic strategies for cancer treatment, potentially disrupting the metastatic potential of tumor cells while controlling cell proliferation. Further research into the multifaceted roles of these genes and their interactions with other signaling pathways will be essential for the advancement of targeted therapies and improved patient outcomes in cancer treatment.

## Precision targeting oncogene-induced cellular plasticity

The complexity of oncogene-induced cellular plasticity, involving the participation of multiple signaling pathways and mechanisms, has made it evident that single-agent approaches may be insufficient to effectively target this phenomenon. For instance, the traditional approach of targeting oncogenes has been to inhibit their activity to reduce cell proliferation rates, thereby controlling tumor growth (Canon et al. 2019). However, the suppression of the tumor cell proliferation phenotype via targeted oncogenes can lead to unintended outcomes, potentially worsening tumor metastasis and related mortality rates (Liu et al. 2012). Moreover, the maintenance of stable tumor cell proliferation may initially appear to suppress tumor metastasis, the excessive proliferation of tumor cells invariably results in an inadequate energy supply and fosters a hostile tumor microenvironment characterized by conditions like hypoxia (Chen et al. 2023). Ultimately, this phenomenon significantly impedes tumor proliferation and subsequently precipitates tumor metastasis. This realization has prompted a shift in cancer therapy towards a more nuanced and multifaceted approach—precision targeting oncogene-induced cellular plasticity—that can effectively target oncogenes without exacerbating metastatic potential.

**Fig. 4:**
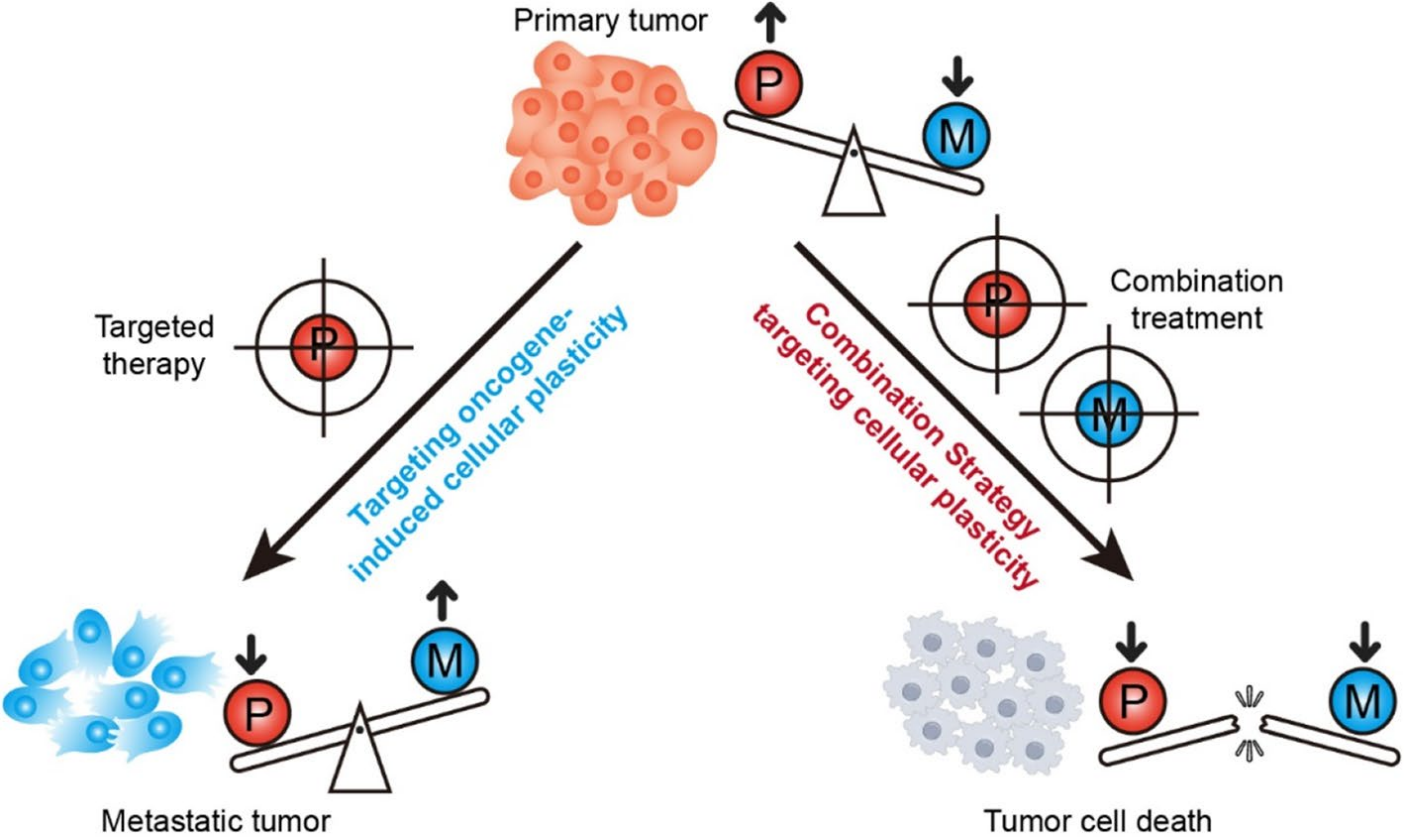
Combination treatments for overcoming the targeting oncogene-induced cellular plasticity

Precision targeting oncogene-induced cellular plasticity involves the simultaneous or sequential use of two or more therapeutic agents with complementary mechanisms of action. By doing so, these strategies aim to maximize therapeutic efficacy while minimizing adverse effects. In the context of dual-role oncogenes, precision targeting oncogene-induced cellular plasticity can be tailored to inhibit the proliferative effects of these genes while preserving or even enhancing their metastatic-suppressing activity (Fig. 4). Although there are currently no direct therapeutic strategies specifically targeting the plasticity induced by Type III genes as described, the clinical exploration of combined MEK (mitogen-activated protein kinase kinase) and FAK (Focal Adhesion Kinase) inhibitors illustrates how this strategy could be implemented. (Banerjee et al. 2021). Clinical trials are currently investigating the effects of MEK inhibitors, which target the proliferative capacity of tumor cells, in conjunction with FAK inhibitors, which aim to reduce metastatic potential. This approach is particularly relevant because some studies have indicated that inhibiting MEK can activate FAK, potentially promoting metastasis (Dawson et al. 2021; Paradis et al. 2021). By combining these inhibitors, we can counteract this unintended consequence and maintain a balance that prevents both uncontrolled proliferation and metastatic spread.

The rationale behind precision therapies for targeting oncogene-induced cellular plasticity is multifaceted. Firstly, cancer cells can exploit different plasticity mechanisms, such as epigenetic regulation, transcriptional regulation, metabolic reprogramming, and stem cell pathways, simultaneously or sequentially, enabling them to adapt and survive under various selective pressures. By combining agents that target these distinct plasticity pathways, researchers aim to comprehensively disrupt the plasticity of cancer cells and limit their ability to develop metastasis. Secondly, tumors are highly heterogeneous, with distinct subpopulations of cancer cells exhibiting different plasticity phenotypes. Precision therapies targeting multiple aspects of cellular plasticity may be more effective in addressing this heterogeneity, reducing the likelihood of treatment-induce metastasis subpopulations emerging. Thirdly, certain combinations of agents targeting different plasticity mechanisms may exhibit synergistic effects, leading to enhanced therapeutic efficacy compared to either agent alone. By exploiting these synergies, precision therapies targeting cellular plasticity may achieve more potent anti-tumor activity while potentially reducing the risk of toxicity associated with higher doses of individual agents. Finally, precision targeting oncogene-induced cellular plasticity can potentially prevent or delay the development of metastasis mechanisms by simultaneously targeting multiple vulnerabilities in cancer cells. This strategy aims to reduce the likelihood of cancer cells acquiring metastasis through compensatory mechanisms or the selection of pre-existing metastasis subpopulations.

In conclusion, the complexity of cellular plasticity in cancer treatment has made single-target approaches insufficient, and precision targeting oncogene-induced cellular plasticity have emerged as a more promising strategy. By comprehensively disrupting plasticity pathways, addressing tumor heterogeneity, leveraging synergistic effects, and preventing or delaying resistance, precision targeting oncogene-induced cellular plasticity hold the potential to achieve better therapeutic outcomes.

## Challenges and perspectives

Precision medicine represents a paradigm that customizes medical interventions according to individual variances and disease attributes, with targeted therapy constituting a pivotal facet of this framework (Collins et al., 2015). Targeted therapy involves the precise targeting of molecular entities implicated in the disease pathology, aiming to optimize therapeutic efficacy while mitigating adverse effects. Nonetheless, the phenomenon of tumor cell plasticity engenders target drift during targeted therapy, whereby the originally identified molecular targets undergo alterations for diverse reasons during treatment, such as in oncogene-targeting therapy, thus influencing therapeutic outcomes. Therefore, adopting a precision targeting strategy for oncogene-induced cellular plasticity, which simultaneously targets both the original target and its potential drifting targets, will be crucial for developing the next generation of targeted therapy techniques.

Despite the promise of precision therapies in targeting oncogene-induced cell plasticity, there are several challenges and considerations that must be addressed to fully realize their potential. Firstly, it requires a deep understanding of the molecular pathways involved in regulating both cell proliferation and metastasis. Comprehensive elucidation of the complex signaling cascades and feedback loops governing these two key hallmarks of cancer is essential. Only through in-depth mechanistic studies can researchers identify the critical nodes that can be effectively targeted in combination. Moreover, the functions of these oncogenes appear to be context-dependent. For instance, in colorectal cancer, CREB1 activates *MYC* through lncRNA CCAT1 (Li et al. 2022), primarily promoting cell proliferation without affecting the regulation of integrin proteins and their associated suppression of metastasis (Liu et al. 2012).

Secondly, the heterogeneity of cancer, both between and within tumors, adds another layer of complexity to the design and implementation of precision therapies. The application of single-cell genomics, transcriptomics, and proteomics can provide valuable insights into the heterogeneity of plasticity phenotypes within a tumor. By identifying and characterizing specific subpopulations exhibiting different plasticity states, researchers can develop targeted strategies tailored to each subpopulation.

The future of cancer treatment lies in the continued exploration of precision therapies and the development of novel strategies to address the challenges posed by oncogene-induced cellular plasticity. Advances in genomics, proteomics, and bioinformatics will be instrumental in identifying new therapeutic targets and understanding the complex interplay between oncogenes and cellular plasticity pathways. Personalized medicine approaches, tailored to individual patient profiles, will also play a significant role in optimizing combination therapies, potentially leading to improved patient outcomes and a new era in cancer treatment.

During the primary tumor stage, tumor cells exhibit robust proliferative capacity but limited metastatic potential. As the tumor cells proliferate, due to factors such as intrinsic genetic mutations in tumor cells, changes in the extrinsic tumor microenvironment, and drugs targeting oncogenes, some tumor cells develop stem-like properties, undergo EMT, metabolic reprogramming, or enter dormancy through plasticity regulation. Consequently, the proliferative capacity of these tumor cells diminishes while their metastatic potential is augmented. Following vascular invasion, these plasticity-regulated cells infiltrate the bloodstream and seed metastatic tumor foci in distant tissues.

Based on the pattern of genes simultaneously regulating cell proliferation and metastasis, genes can be classified into four types: Type I, genes that concurrently promote proliferation and metastasis, such as *HIF1a*, *EGFR*, and *KRAS*; Type II, genes that simultaneously inhibit proliferation and metastasis, such as *TP53*, *PTEN*, and *RB*; Type III, genes that promote proliferation but inhibit metastasis, such as *CREB1*, *MYC*, and *PHGDH*; Type IV, genes that inhibit proliferation but promote metastasis, such as *TGF-β*, *SNAIL* and *CDKN1A*.

Type III genes facilitate cell proliferation while concurrently restraining cell metastasis. For instance, *CRBE1, FBXO22, TMEM16A, SnoN,* and *MAZ* bolster cell proliferation by fostering the cell cycle progression, yet they concurrently hinder cell metastasis by impeding the EMT process. Additionally, *MYC* and *PHGDH* regulate cell proliferation capacity (P) by governing the cell cycle and serine synthesis, respectively. Simultaneously, they transcriptionally suppress integrin proteins, diminishing cell adhesion to the extracellular matrix and fostering cell metastatic potential (M).

Initially, primary tumor cells exhibit robust proliferative capacity (P), yet their metastatic potential (M) remains relatively constrained. Targeted therapy aimed at inhibiting oncogenes can partially curb the proliferative capacity of tumor cells. However, due to tumor cell plasticity, certain cells undergo EMT, metabolic reprogramming, and other alterations, thereby augmenting their metastatic potential and culminating in the development of metastatic tumors. Consequently, solely targeting tumor cell proliferation with such therapies may prove inadequate for complete cancer eradication. Combination therapy endeavors to concurrently address pivotal drivers of induced cellular plasticity, including oncogenes, EMT transcription factors, and metabolic enzymes. By collectively targeting these critical factors, it is anticipated to more effectively suppress both tumor cell proliferation and metastatic potential, ultimately precipitating tumor cell demise.

## Data Availability

Not applicable.
